# Comparative Genomics Reveals Evolutionary Constraints of Regulatory Elements in the *Sus scrofa* Genome

**DOI:** 10.3390/ani16091296

**Published:** 2026-04-23

**Authors:** Mingfang Zhou, Huashui Ai

**Affiliations:** National Key Laboratory for Swine Genetic Improvement and Germplasm Innovation, Ministry of Science and Technology of China, Jiangxi Agricultural University, Nanchang 330045, China; duzhoumingfang@hotmail.com

**Keywords:** *Sus scrofa*, comparative genomics, evolutionary constraint, regulatory elements, transposable element

## Abstract

REs play critical roles in modulating gene expression and shaping phenotypic diversity; however, their evolutionary dynamics in pigs remain incompletely understood. Based on genome alignments across 13 species, we characterized the hierarchical structure of REs evolution in pigs, consisting of cross-species conserved, Suidae-specific, and *Sus scrofa*-specific regulatory elements. Only 2.86% of REs were conserved across species, whereas 16.35% were Suidae-specific, suggesting lineage-specific regulatory innovation in pigs. Cross-species constrained REs exhibited higher conservation and were preferentially located proximal to transcription start sites. *Sus scrofa*-specific REs exhibited lower conservation, higher TE content, and were mainly distributed in intergenic regions. These patterns suggest that TEs may contribute to regulatory sequence turnover during pig evolution. Functional enrichment analysis showed that conserved REs were associated with fundamental developmental and neurobiological processes, whereas *Sus scrofa*-specific REs were enriched in pathways related to lipid metabolism, immune response, and neural signaling. In addition, intra-species analysis across 35 pig genomes demonstrated high overall conservation of REs. Our study reveals distinct evolutionary patterns of regulatory elements in pigs and provide novel insights into regulatory innovation underlying phenotypic diversity and economically important traits in livestock.

## Simple Summary

In this study, we systematically analyzed the evolutionary constraints and innovation patterns of pig regulatory elements (REs) across different evolutionary scales. REs proximal to the transcription start site (TSS) exhibited stronger evolutionary constraint, whereas *Sus scrofa*-specific REs were predominantly located in noncoding regions and exhibited a higher proportion of transposable elements (TEs), suggesting that TEs may be involved in lineage-specific REs. Functionally, cross-species constrained REs are enriched in pathways related to development and morphogenesis, which are conserved biological processes. Meanwhile, *Sus scrofa*-specific REs are associated with lipid metabolism, stress response, immune defense, and neural signaling pathways, indicating their potential involvement in lineage-specific regulatory changes. Furthermore, REs maintain high stability across pig populations and show a conserved regulatory landscape at the intraspecific level.

## 1. Introduction

The precise regulation of gene expression represents a fundamental mechanism underlying the formation of complex biological traits, developmental processes, and adaptive evolution of species. Regulatory elements (REs) orchestrate the spatiotemporal expression patterns of genes through interactions with transcription factors and participation in the establishment of chromatin architecture. Accumulating evidence indicates that mutations within regulatory regions constitute a major source of phenotypic variation across organisms [1]. Therefore, understanding the conservation and diversification of REs is important for explaining phenotypic diversity and species divergence.

Recent advances in epigenomics and comparative genomics have substantially expanded our understanding of the function and evolution of regulatory elements. The Encyclopedia of DNA Elements (ENCODE) project has systematically mapped human regulatory elements by integrating data on chromatin accessibility, histone modifications, DNA methylation, and transcription factor binding, identifying nearly one million candidate cis-regulatory elements (cCREs) [2]. Comparative genomic analyses based on 241 mammalian genomes have quantitatively assessed the evolutionary constraints acting on cCREs. Approximately half of human cCREs were found to be under evolutionary constraint across mammals, and distinct evolutionary patterns were observed among different element types (e.g., promoter-like and enhancer-like elements). Notably, primate-specific cCREs were found to be significantly enriched in genes involved in environmental response [3]. Beyond mammals, similar approaches in plants, particularly in maize, have revealed that TEs contribute extensively to the formation and remodeling of open chromatin regions, driving intra-species regulatory innovation and gene expression variation [4]. These multi-genome comparative studies reveal patterns of conservation and lineage-specific changes in regulatory sequences across species.

In livestock species, particularly domestic animals, significant progress has been made in the functional annotation and evolutionary analysis of regulatory elements. The Functional Annotation of Animal Genomes (FAANG) consortium aims to generate comprehensive maps of functional elements in domesticated animals, enabling systematic annotation of regulatory elements in pigs, cattle, and chickens [5]. Additionally, comparative epigenomic studies among humans, pigs, and mice have revealed unexpectedly high conservation of regulatory elements between pigs and humans, with the proportion of shared elements substantially exceeding that observed between humans and mice [6]. This suggests that some pig regulatory elements may retain conserved regulatory functions across mammals. Although pig regulatory elements have been investigated to some extent, a systematic cross-species comparative perspective is still lacking. In particular, it remains unclear which porcine regulatory elements are deeply conserved across mammals, which are restricted to the Suidae lineage, and which are specific to *Sus scrofa*, as well as how these distinct evolutionary patterns have shaped the pig regulatory genome.

In this study, we integrated multi-tissue pig regulatory element data from the FAANG project with cross-species genome alignments and publicly available population genomic data to investigate the evolutionary patterns of porcine regulatory elements at both interspecific and intraspecific levels. Our analyses provide a hierarchical view of regulatory element evolution in pigs and clarify how different classes of regulatory elements vary across evolutionary levels, with implications for both evolutionary biology and livestock genetics.

## 2. Materials and Methods

### 2.1. Functional Enrichment Analysis of Genes Associated with Pig Regulatory Elements

In this study, we utilized the cis-regulatory elements identified across eight pig tissues provided by the FAANG [5]. Classification of REs followed the annotation criteria defined by the FAANG consortium. According to genomic locations, REs were categorized into three classes: (1) TSS-proximal REs, regions located within ≤2 kb of the transcription start site (TSS) of protein-coding genes; (2) Genic REs, regions overlapping gene bodies but outside the TSS ± 2 kb window; and (3) Intergenic REs, regions that overlap neither gene bodies nor the TSS ± 2 kb window. REs were subsequently assigned to their nearest genes based on their genomic proximity. Functional enrichment analysis was performed using Metascape v3.5 [7] with default parameters.

### 2.2. Evolutionary Constraint Analysis

The genome assemblies used in this study were downloaded from the National Center for Biotechnology Information (NCBI, https://www.ncbi.nlm.nih.gov/, accessed on 1 September 2025) and the National Genomics Data Center (NGDC, https://ngdc.cncb.ac.cn/, accessed on 1 September 2025). A total of 47 genome assemblies were included in this analysis. Of them, 13 genomes were used for interspecific constraint analysis, encompassing important members of the Suidae family, Catagonus wagneri as the outgroup, as well as human, mouse (a model organism), and other economically important domesticated animal species (Table S1). For the intraspecific constraint analysis, 35 pig genome assemblies, including the reference genome, were used. These assemblies represent different breeds of domestic pigs from both European and Asian populations, as well as wild boar genomes, all of which belong to the species *Sus scrofa* (Table S2).

To evaluate interspecific and intraspecific evolutionary constraints, we independently aligned the reference genome with each of the other species using Cactus v2.6.8 [8], and pairwise alignment chains were subsequently extracted via the cactus-hal2chains utility. Using the resulting pairwise alignments, we assessed the conservation of REs with the liftOver utility provided by UCSC Genome Browser (http://genome.ucsc.edu, accessed on 1 September 2025). Each RE in the reference genome was projected onto the genomes of the other species, and its alignment success was quantified. Specifically, N1 denotes the number of species in which ≥90% of the nucleotide sequence within the RE could be aligned to the reference genome, while N2 denotes the number of species in which ≤10% of the RE’s nucleotides could be aligned. The distribution patterns of N1 and N2 were then used to characterize evolutionary constraints acting on REs. Elevated N1 values indicate high levels of sequence conservation, suggesting evolutionary constraint, while high N2 values reflect poor alignment success and thus denote lineage-specific evolutionary divergence.

For regions phyloP and PhastCons score calculation, multiple sequence alignments of interspecies genomes were generated using Cactus v2.6.8 [8]. Scores were then computed with the PHAST v1.4 package [9], including phyloFit [10], phastCons [11], and phyloP [12]. PhyloFit constructs a neutral evolutionary model. PhastCons scores were obtained using the phastCons utility with parameters: target coverage = 0.3, expected length = 45, rho = 0.31. PhyloP scores were obtained using the phyloP utility with the LRT method, branch test, and CONACC mode. PhyloP scores greater than 0 indicate evolutionary conservation, whereas scores less than 0 indicate accelerated evolution. PhastCons evaluates the degree of evolutionary conservation, with higher values indicating stronger conservation.

### 2.3. Annotation of Repetitive Elements in the Porcine Reference Genome

Repetitive sequences in the Sscrofa 11.1 reference genome were annotated using RepeatMasker v4.1.0 (https://www.repeatmasker.org/, accessed on 30 October 2019) in combination with the Dfam-3.1 [13] and RepBase-20181026 [14] databases. Default parameters were employed for the analysis, and all repetitive elements were automatically classified into their corresponding repeat families.

### 2.4. Prediction of Transcription Factor Motifs

To systematically identify potential transcription factor binding sites in the porcine genome, we used FIMO v5.5.8 [15] with the vertebrate core non-redundant motif set from JASPAR 2022 [16]. The *Sus scrofa* 11.1 reference genome was scanned using a statistical significance threshold of *p* < 1 × 10^−6^.

### 2.5. Dimensionality Reduction Analysis

The liftOver utility from the UCSC Genome Browser (http://genome.ucsc.edu, accessed on 1 September 2025) was used to calculate the proportion of aligned bases for each RE across the genomes of other species, generating a conservation matrix. Principal component analysis (PCA) was subsequently performed to reduce the dimensionality of these high-dimensional conservation features for visualization.

### 2.6. Hi-C Analysis

Two Hi-C replicates of porcine embryonic fibroblasts (PEFs) were downloaded from the Gene Expression Omnibus (GEO) database under the accession number GSE153452. Then the data were merged and analyzed for topologically associating domains (TADs) and chromatin loops. For TAD analysis, HiCExplorer v3.72 [17] was employed at a resolution of 25 kb. Regarding chromatin loop detection, Mustache v1.01 [18] was utilized with a resolution of 5 kb and a significance threshold of *p* ≤ 0.05. Corresponding histone modification peaks were also retrieved from the same dataset.

### 2.7. Overlap Analysis Between QTLs

Pig QTLs were downloaded from the Animal Quantitative Trait Loci Database (Animal QTLdb, https://www.animalgenome.org/cgi-bin/QTLdb/index, release56, accessed on 25 April 2025). To avoid potential bias, QTLs larger than 1 Mb were excluded from the analysis.

## 3. Results

### 3.1. Evolutionary Constraint Analysis of REs Across Species

**Figure 1:**
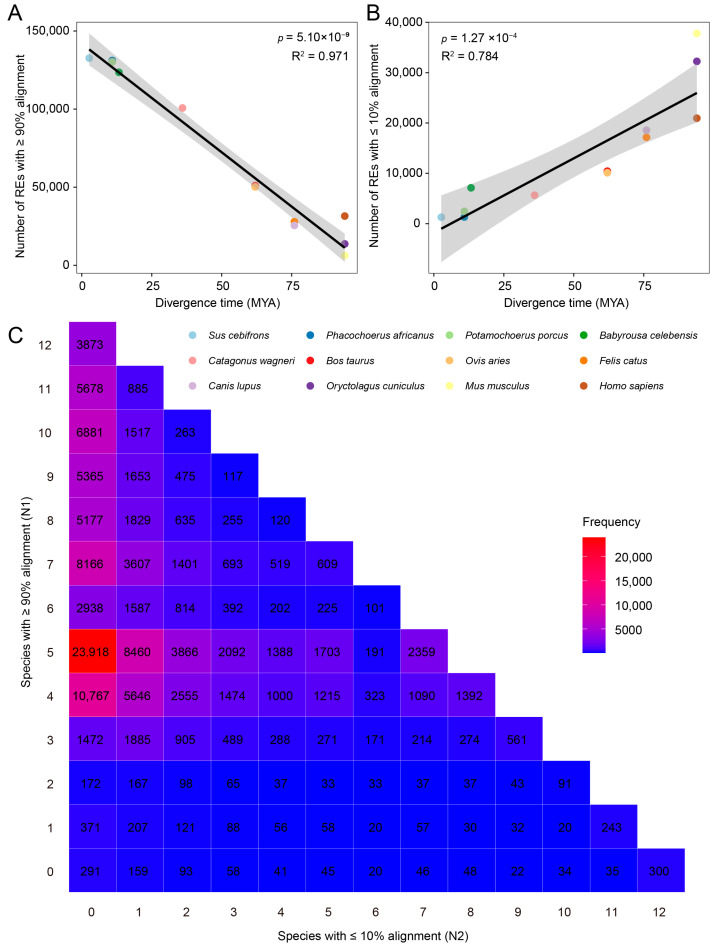
Relationship between inter-species alignment of pig regulatory elements (REs) and evolutionary divergence time. (**A**) Linear regression of the number of REs with ≥90% alignment against divergence time. (**B**) Linear regression of the number of REs with ≤10% alignment against divergence time. MYA: million years ago. (**C**) Inter-species evolutionary constraint distribution. N1 denotes the number of genomes in which ≥90% of REs can be aligned, and N2 denotes the number of genomes in which ≤10% of REs can be aligned. Higher N1 values indicate conservation across a greater number of species, whereas higher N2 values indicate lower conservation across species and greater lineage-specific divergence.

Based on 135,254 cross-tissue aggregated REs from pigs provided by the FAANG project, we performed cross-species alignment and sequence conservation analysis. Using the Sscrofa11.1 genome as the reference, we aligned these REs to the genomes of 12 other mammalian species and quantified the number of REs that could be mapped with high (≥90%) and low (≤10%) sequence identity in each genome. Linear regression analysis integrating species divergence times from the TimeTree (https://timetree.org/) revealed that the number of REs with ≥90% alignment identity significantly decreased with increasing divergence time (*p* = 5.10 × 10^−9^, R^2^ = 0.971; Figure 1A, Supplementary Table S1). Conversely, the number of REs with ≤10% alignment identity significantly increased with divergence time (*p* = 1.27 × 10^−4^, R^2^ = 0.784; Figure 1B). These results demonstrate that the sequence conservation of pig REs across species is strongly correlated with phylogenetic distance.

The cross-species mapping results of pig REs across 12 other mammalian species are illustrated in a heatmap (Figure 1C). In total, we identified 3873 cross-species constrained REs (successfully mapped across all examined species), accounting for 2.86% of all REs; 300 *Sus scrofa*-specific REs (unmappable to all non-pig species), representing 0.22% of the total; and 22,123 Suidae-specific REs (successfully mapped across all Suidae species but not in non-Suidae species), constituting 16.35% of all REs, which is the highest proportion among all categories. These results show that pig REs exhibit high sequence conservation within Suidae lineages but lower consistency in mammalian species more distantly related to Suidae.

**Figure 2:**
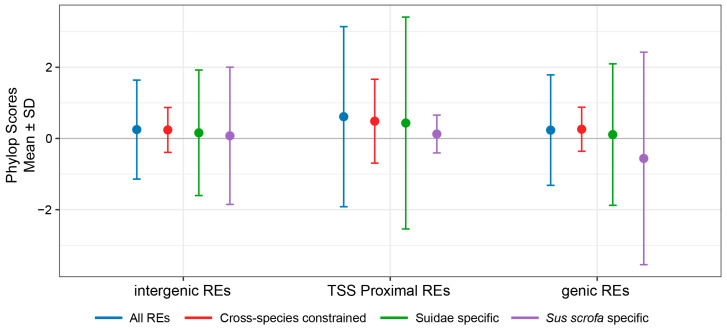
PhyloP scores (Mean ± SD) for different types of REs.

To evaluate the evolutionary conservation of different categories of REs, we use phastCons and phyloP scores to measure (Figure 2, Figures S1 and S2). Cross-species conserved REs generally exhibited higher phyloP scores compared to Suidae-specific and *Sus scrofa*-specific REs. By contrast, *Sus scrofa*-specific REs had the lowest phyloP scores, suggesting these elements had the lowest level of conservation in the pig lineage. Based on the genomic annotations of different REs from the FAANG project [5], we found that TSS-proximal REs had the highest conservation level, while genic REs exhibited the lowest (Figure 2 and Figure S1). Within *Sus scrofa*-specific REs, genic REs had the lowest phyloP scores, indicating that regulatory elements within gene bodies may have undergone more rapid evolutionary divergence. PhastCons scores followed a similar trend, with cross-species conserved REs exhibiting the highest values, consistent with weaker conservation in lineage-specific elements (Figure S2).

Collectively, these findings support that the level of evolutionary constraint on pig REs is closely associated with their genomic localization. REs adjacent to the TSS are more evolutionarily conserved, while gene bodies may harbor more lineage-specific regulatory innovations.

### 3.2. Distribution Characteristics of Inter-Species Constrained REs

**Figure 3:**
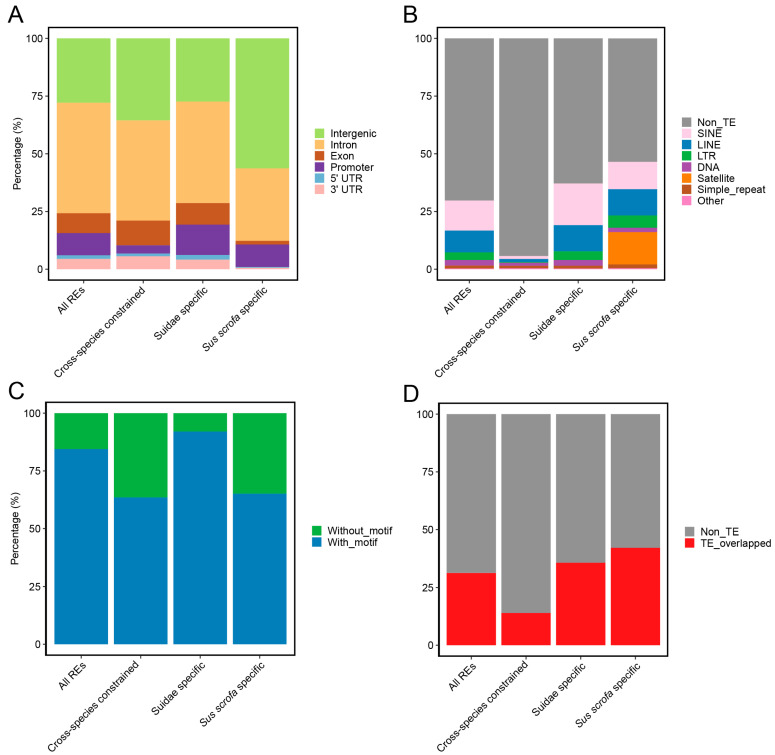
Distributional characteristics of interspecies-constrained REs. (**A**) Distribution of constrained REs in genomic features. (**B**) Proportion of TE in constrained REs. (**C**) Proportion of REs with motif. (**D**) Proportion of motif overlapping TEs.

We compared the distribution characteristics of REs with different levels of inter-species constraint. Analysis of their genomic location distribution showed that REs in all groups were primarily located in intronic and intergenic regions (Figure 3A). Among these, *Sus scrofa*-specific REs exhibited the highest proportion in intergenic regions (56.33%); Suidae-specific REs had a relatively higher proportion in promoter regions (13.12%); whereas cross-species constrained REs showed a higher proportion located in exonic regions compared to the other groups.

In the TE coverage analysis (Figure 3B), TE sequences accounted for 29.74% of the total length across all REs. Cross-species constrained REs displayed the lowest TE coverage (0.56%), while *Sus scrofa*-specific REs exhibited the highest TE coverage (46.51%). Regarding TE class composition, *Sus scrofa*-specific REs showed higher proportions of LINEs, LTRs, Satellites, and Simple repeats; conversely, the overall proportions of various TE classes were relatively low in cross-species constrained REs.

By integrating the motif analysis across the genome, the results (Figure 3C) indicated that Suidae-specific REs had the highest proportion of motifs, consistent with their higher distribution in promoter regions. Further analysis of the overlap between motifs and TEs within REs revealed a trend consistent with the TE proportion results: *Sus scrofa*-specific REs showed the highest overlap proportion, followed by Suidae-specific REs, and cross-species constrained REs the lowest (Figure 3B,D).

In summary, *Sus scrofa*-specific REs are primarily distributed in intergenic regions and possess the highest TE coverage, suggesting that these regions may represent potential sources of lineage-specific regulatory variation, possibly associated with TE. Conversely, the lowest TE proportion in cross-species constrained REs indicates that these highly conserved regulatory elements across different species are less influenced by transposable elements, consistent with potentially conserved regulatory functions across species.

### 3.3. Functional and Potential Trait Associations of REs

**Figure 4:**
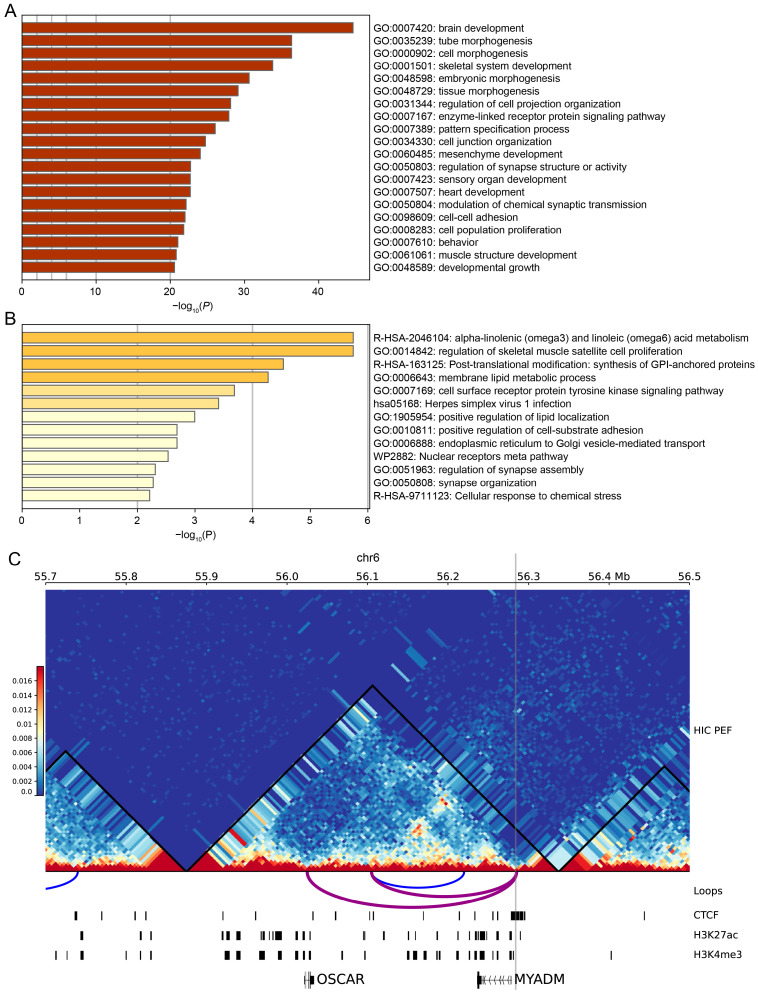
Functional of genes related to REs. (**A**) Functional enrichment of genes related to cross-species constrained REs. (**B**) Functional enrichment of genes related to *Sus scrofa*-specific REs. Darker bar colors indicate higher statistical significance in each panel. (**C**) Example of three-dimensional chromatin regulation involving a *Sus scrofa*-specific RE, the gray vertical shading indicates the position of the RE, while the purple arc represents the loop whose anchor coincides with the RE.

Using a gene proximity-based annotation strategy, cross-species constrained REs and *Sus scrofa*-specific constrained REs were assigned to 2,764 and 179 putative target genes. Functional enrichment analyses revealed a marked functional partitioning between cross-species constrained and *Sus scrofa*-specific REs (Figure 4A,B; Tables S3 and S4). Cross-species constrained REs were predominantly enriched in developmental and morphogenetic programs, including neurodevelopment, organogenesis, and multiple layers of tissue morphogenesis (Figure 4A; Table S3). These elements were also significantly associated with synaptic organization and modulation of chemical synaptic transmission. In contrast, *Sus scrofa*-specific REs were enriched in lipid metabolic processes, membrane-related functions, intracellular transport, and signaling pathways (Figure 4B; Table S4). Enriched terms included alpha-linolenic and linoleic acid metabolism, membrane lipid metabolic process, positive regulation of lipid localization, GPI-anchored protein synthesis, and cellular response to chemical stress, as well as a viral infection-associated pathway.

To explore the regulatory potential of *Sus scrofa*-specific REs, we analyzed 3D chromatin structure data from pig embryonic fibroblasts. On chromosome 6, a *Sus scrofa*-specific RE is connected to the *OSCAR* gene via a chromatin loop and is located within the same TAD (Figure 4C). Meanwhile, the specific RE overlaps with both LINE elements (Table S5) and CTCF peaks (Figure 4C), suggesting potential involvement in TE-associated regulatory architecture and chromatin organization. *OSCAR* encodes an immunoglobulin-like receptor involved in modulating both innate and adaptive immune responses [19]. Transcriptome data from the PigGTEx database [20] reveals high expression of the *OSCAR* gene in blood, an immune-related tissue (Figure S3). Furthermore, we integrated the pigQTL database and identified eight Sus scrofa-specific REs overlapping with QTLs associated with economically important traits, including reproduction, growth, and meat quality (Table S6). These observations suggest that Sus scrofa-specific REs are associated with immune-related genomic regions in pigs, though further functional validation is required.

### 3.4. Intra-Species Evolutionary Constraint Analysis

**Figure 5:**
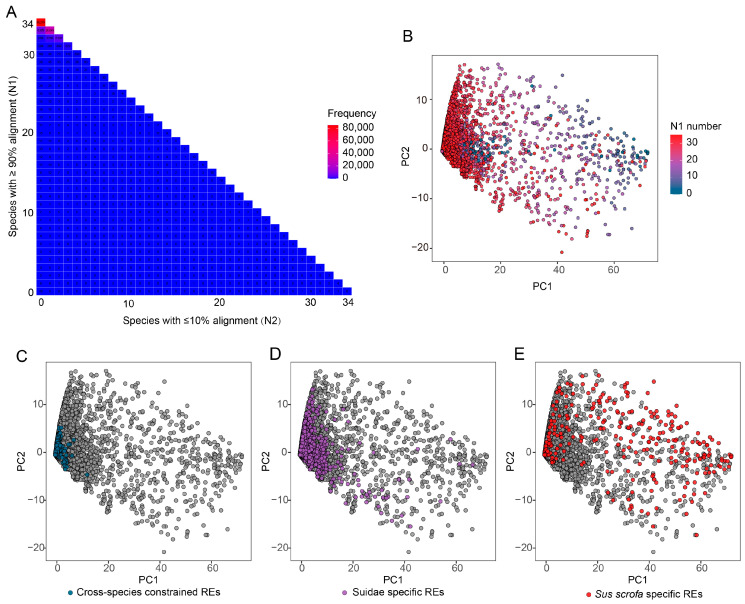
Intra-species distribution of evolutionary constraint of REs. (**A**) Intra-species evolutionary constraint distribution. N1 denotes the number of genomes in which ≥90% of REs are alignable, and N2 denotes the number of genomes in which ≤10% of REs can be aligned. (**B**) PCA plot based on the proportion of alignable bases of each RE across other genomes. (**C**–**E**) PCA visualization of REs within species at different evolutionary levels. The distribution patterns reflect the degree of consistency in RE presence across genomes, with tighter clustering indicating REs that are consistently present across most genomes, and more dispersed patterns indicating REs that are inconsistently present and exhibit higher variability among genomes.

During domestication processes, pigs have formed diverse breeds in different geographic regions. Based on the pig RE dataset provided by the FAANG project, we further analyzed the constraint levels of these REs within the pig species, examining a total of 34 genomes in addition to the reference genome, including representative European domestic pigs, Asian domestic pigs, and European wild boars (Table S2). Alignment results show that REs are highly conserved in most genomes (Figure 5A). Specifically, 86,219 REs (63.7% of total REs) were successfully mapped in all pig genomes; 133,545 REs (98.7% of total REs) were successfully mapped in at least 30 genomes, demonstrating the high level of conservation of REs at the intraspecific level. Principal component analysis based on the proportion of alignable bases for each intra-species RE (Figure 5B) showed no distinct clustering among different pig populations, further supporting the high overall consistency of REs within the species. REs alignable in more genomes tended to cluster on the left side of the plot, while those with lower alignment success were distributed on the right side, exhibiting greater dispersion.

To investigate the differences among REs from distinct evolutionary levels, we performed dimensionality reduction on the alignment rate matrix data for cross-species constrained REs, Suidae-specific REs, and *Sus scrofa*-specific REs. The results showed that cross-species constrained REs clustered on the left side of the plot, indicating high conservation (Figure 5C); Suidae-specific REs showed moderate dispersion (Figure 5D); and *Sus scrofa*-specific REs, despite being the fewest in number, exhibited the most scattered distribution (Figure 5E). These results suggest that *Sus scrofa*-specific elements show high levels of variation within populations, consistent with weaker evolutionary constraint.

## 4. Discussion

In this study, we systematically characterized the evolutionary landscape of REs in pigs across multiple phylogenetic scales. The hierarchical patterns of REs show that the porcine regulatory genome has undergone both deep conservation and lineage-specific remodeling during mammalian evolution. Only a small fraction of REs were deeply conserved across all examined species. In contrast, a considerable proportion were Suidae specific, consistent with substantial lineage-specific regulatory innovation during pig evolution. Similar patterns of limited cross-species conservation have been reported in plants, where only a small percentage of maize open chromatin regions are shared across Poaceae species [4]. By comparison, a much higher proportion of human candidate cis-regulatory elements are conserved across mammals [3], which indicates lineage-dependent differences in regulatory constraint. Although all genomes analyzed in this study are mammalian, the proportion of conserved REs was substantially lower than that reported for human candidate cis-regulatory elements across mammals. This discrepancy may result from lineage-specific variation in regulatory sequences and may also reflect differences in the number of species analyzed and their sample sizes. Overall, our study provides a comprehensive, multi-scale view of the porcine regulatory genome. We integrated cross-species conservation, TE content, and 3D chromatin interactions to identify both conserved and lineage-specific regulatory elements. These elements likely contribute to phenotypic diversity in pigs.

The degree of constraint varied among different RE categories. Cross-species constrained REs exhibited the highest levels of conservation, followed by Suidae-specific and *Sus scrofa*-specific REs, indicating progressively relaxed evolutionary constraint along lineage specificity. Regulatory element conservation also differed across genomic regions. REs located proximal to TSS showed the strongest constraint, whereas genic REs exhibited comparatively weaker conservation. This pattern is consistent with the essential regulatory roles of TSS-proximal regions, which are expected to be subject to stronger purifying selection [21]. In studies of regulatory innovation, TEs play an important role. In plants, TEs have substantially influenced regulatory evolution [22]. In mammals, including pigs, recently evolved (young) TEs have been reported to affect gene regulation, genetic diversity, and complex traits [23]. Our study observed an elevated proportion of TEs in *Sus scrofa*-specific REs. This enrichment suggests a potential contribution of TEs to the emergence of lineage-specific regulatory elements; however, this interpretation remains correlative in the absence of systematic analyses of TE age, activity, and functional impact. Many studies have reported that TEs act as regulatory elements in the pig genome. For instance, a specific SINE insertion within the *MYO5A* transcript has been associated with alternative splicing and coat color diversity in European pig populations [24]. Another study reported that SINE insertions can act as repressive elements to reduce *LEPROT* expression, serving as molecular markers for growth trait selection in pig breeding [25]. Notably, we identified a *Sus scrofa*-specific RE containing a LINE element that exhibits chromatin looping interactions with the immune-related gene *OSCAR*. This three-dimensional genome interaction supports a potential influence of TEs on RE innovation.

Functional enrichment analysis further elucidated the biological processes potentially involving *Sus scrofa*-specific REs. Associated genes were significantly enriched in pathways related to lipid metabolism, chemical stress response, immune defense, and neural signal transduction. These functional categories closely align with metabolic adaptations, stress resistance, and behavioral traits shaped during pig domestication. Notably, immune-related genes have been shown to evolve rapidly in the pig genome [26].

In domestic pigs, which encompass diverse breeds, regulatory element activity varies substantially among breeds, potentially associated with sequence variation [6]. Through analysis of 35 representative pig genomes, we found that REs exhibit remarkably high conservation at the intra-species level. Principal component analysis revealed no clear clustering among different pig populations. This indicates that REs remain highly conserved during intra-species evolution, reflecting the stability of regulatory networks at the species level. REs from different evolutionary levels showed consistent trends within species: cross-species constrained REs were most conserved, Suidae-specific REs exhibited intermediate variation, and *Sus scrofa*-specific REs showed the greatest variation across breeds. These findings suggest that lineage-specific REs remain in a dynamic state of ongoing evolution, accumulating polymorphisms across different breeds. Multiple studies have shown that variation across populations may influence gene expression by affecting regulatory elements and ultimately impacting phenotypic traits [27,28]. Therefore, *Sus scrofa*-specific REs not only reflect historical signatures of lineage divergence but may also contribute to ongoing intra-species adaptive differences.

Despite the multi-layered insights into RE evolution revealed by this study, several limitations should be acknowledged. For example, the limited number of species analyzed precludes a comprehensive understanding of pig RE evolution across a broader phylogenetic context. This study focused mainly on interspecies patterns and did not examine breed-specific regulatory variation or its links to complex traits, which could be addressed in future work using GWAS and eQTL analyses. In addition, our study relied primarily on sequence-based comparative analyses and lacks experimental validation. Future studies integrating CRISPR-based gene editing, transcriptomic analyses, and phenotypic validation will be essential to functionally characterize these lineage-specific REs and elucidate their precise roles in lipid metabolism, immune regulation, and neural adaptation.

## 5. Conclusions

Collectively, our multi-layered analysis reveals distinct evolutionary patterns of porcine REs at both inter- and intra-species levels. Cross-species constrained REs are enriched in developmental pathways, highlighting their essential roles in mammalian evolution, while *Sus scrofa*-specific REs show enrichment in lipid metabolism and immune defense, suggesting they may play a role in lineage-specific regulatory adaptation. Future functional characterization of these elements will facilitate their application in pig genetic improvement.

## Data Availability

All data analyzed in this study are publicly available. The pig regulatory elements identified across eight pig tissues were obtained from the FAANG consortium. The genome assemblies used for evolutionary constraint analysis were downloaded from the NCBI and NGDC databases. The specific genome assemblies and accession numbers used in this study are provided in Supplementary Tables S1 and S2. Hi-C data from porcine embryonic fibroblasts were retrieved from GEO under accession number GSE153452.

## Supplementary Materials

The following supporting information can be downloaded at https://www.mdpi.com/article/10.3390/ani16091296/s1, Figure S1: Distribution of phyloP scores for all REs; Figure S2: Distribution of phastCons scores for all REs; Figure S3: The cross-tissue expression profiles of *OSCAR* in the PigGTEx database; Table S1: Genomes information for interspecies constraint analysis; Table S2: Genomes information for intraspecies constraint analysis; Table S3: Functional enrichment of cross-species constrained REs related genes; Table S4: Functional enrichment of *Sus scrofa*-specific REs related genes.; Table S5: Info about Loop anchor’s RE and TE; Table S6: Overlap between REs and pig QTL.
