# Supplementary figures and images for: Comparative Genomics Reveals Evolutionary Constraints of Regulatory Elements in the Sus scrofa Genome
Source: Animals (Basel). 2026 Apr 23;16(9):1296. doi: 10.3390/ani16091296 (PMC13162708; doi:10.3390/ani16091296)

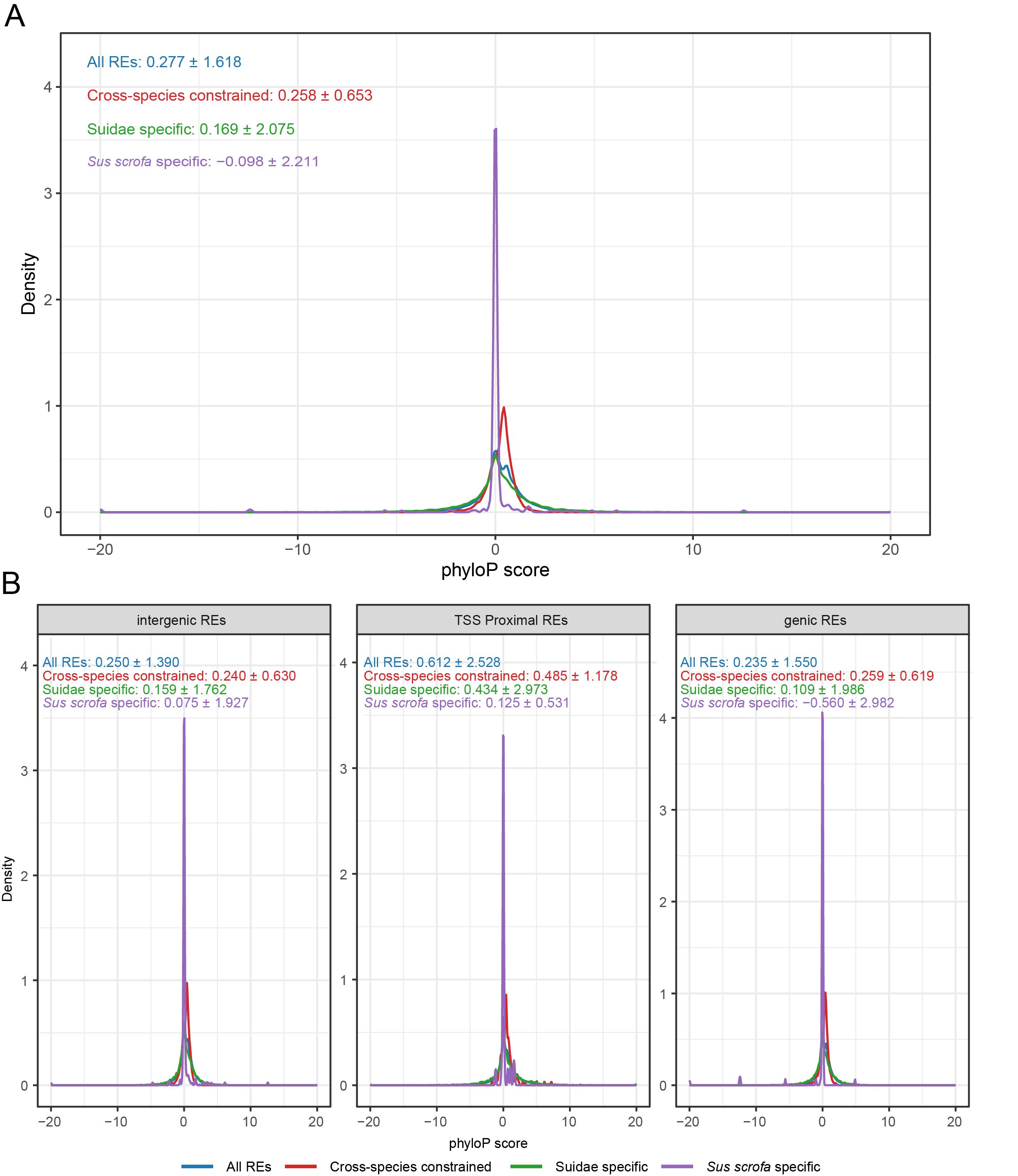

Supplement: Supplementary file 1 [file animals-16-01296-s001.zip › Figure S1.tif]

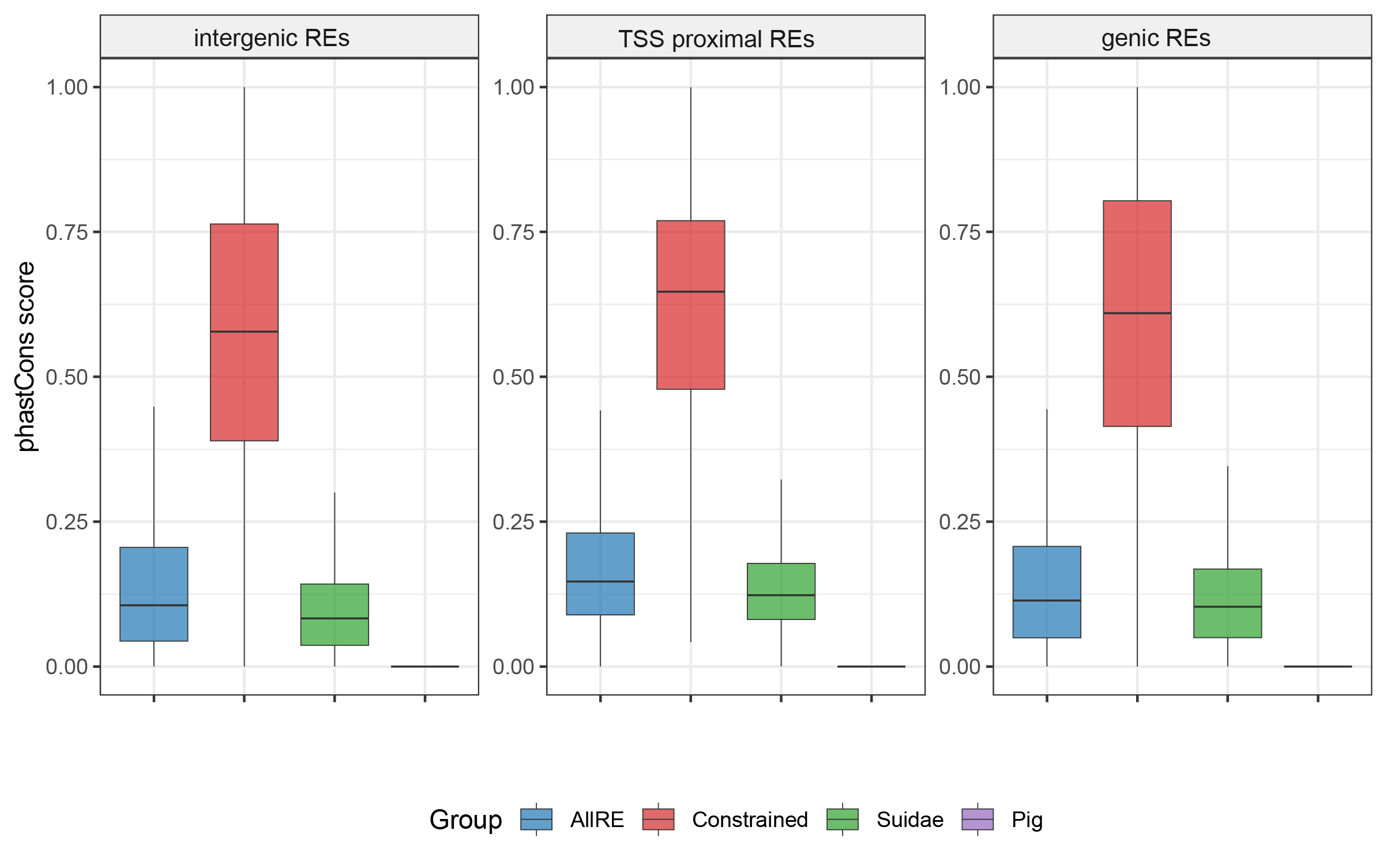

Supplement: Supplementary file 1 [file animals-16-01296-s001.zip › Figure S2.tif]

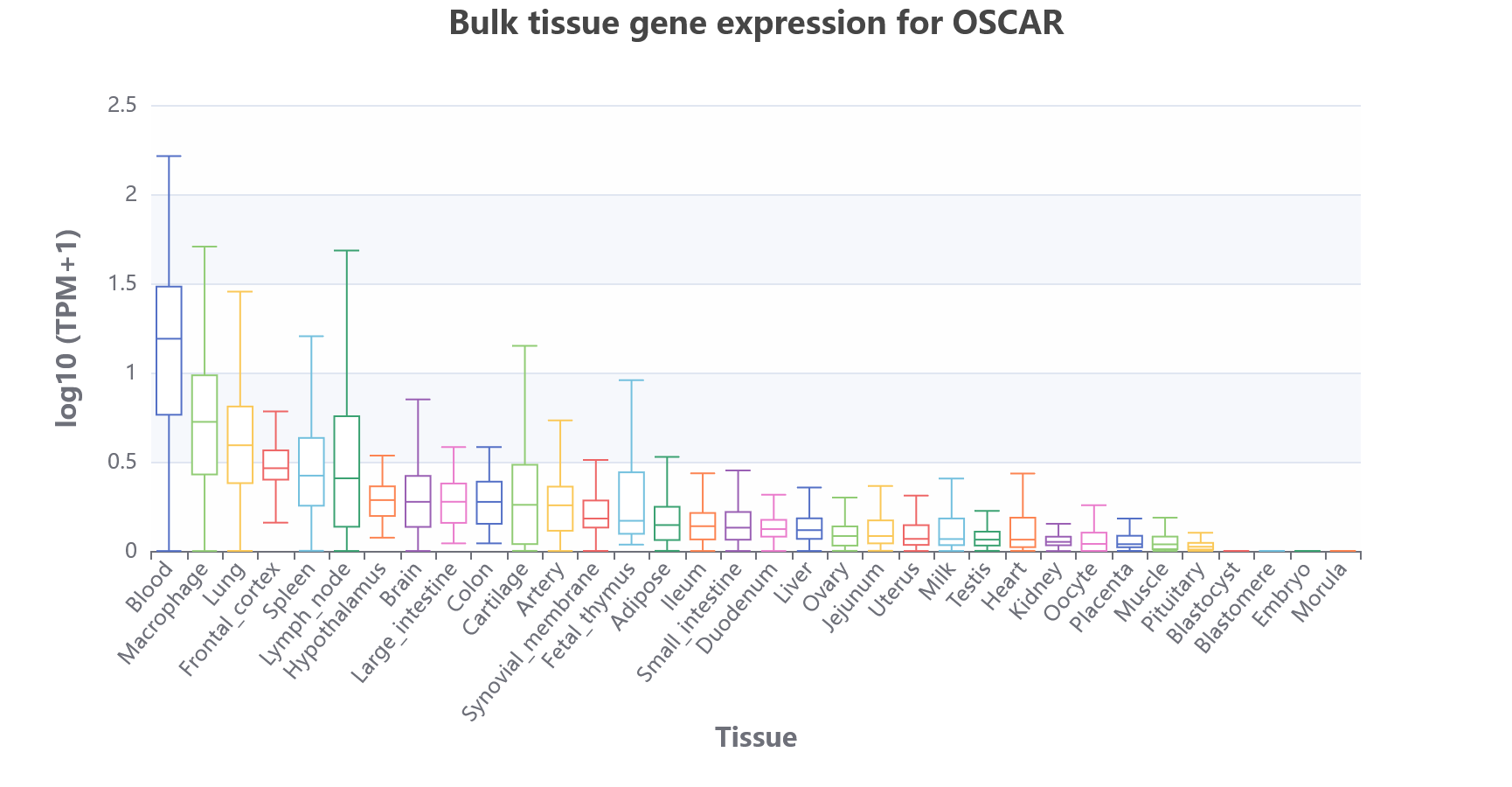

Supplement: Supplementary file 1 [file animals-16-01296-s001.zip › Figure S3.png]
